# Decreased Functional Connectivity of the Primary Visual Cortex and the Correlation With Clinical Features in Patients With Intermittent Exotropia

**DOI:** 10.3389/fneur.2021.638402

**Published:** 2021-03-26

**Authors:** Xueying He, Jie Hong, Zhaohui Liu, Qian Wang, Ting Li, Xiaoxia Qu, Nanxi Fei, Wei Li, Jing Fu

**Affiliations:** ^1^Department of Radiology, Beijing Tongren Hospital, Capital Medical University, Beijing, China; ^2^Department of Ophthalmology, Beijing Tongren Hospital, Capital Medical University, Beijing, China

**Keywords:** intermittent exotropia, functional magnetic resonance imaging, resting state fMRI, spontaneous activity, functional connectivity

## Abstract

The purpose of this study is to investigate characteristic alterations of functional connectivity (FC) patterns in the primary visual area (V1) in patients with intermittent exotropia (IXT) using resting-state functional magnetic resonance imaging (rs-fMRI) and how they relate to clinical features. Twenty-six IXT patients and 21 age-, sex-, handedness-, and education-matched healthy controls (HCs) underwent rs-fMRI. We performed FC analyses between bilateral V1 and other brain areas and compared FC strength between two groups. A Pearson correlation analysis was used to evaluate the correlation between the FC differences and clinical features. Compared with HCs, patients with IXT showed significantly lower FC of the right V1 with the right calcarine sulcus and right superior occipital gyrus, and the left V1 with right cuneus and right postcentral gyrus. The Newcastle Control Test score was positively correlated with mean FC values between the left inferior parietal lobule and bilateral V1, and between the left supramarginal gyrus and left V1. The duration of IXT was positively correlated with mean FC values between the right inferior occipital gyrus and right V1. Reduced FC between the V1 and various brain regions involved in vision and eye movement processes may be associated with the underlying neural mechanisms of impaired visual function in patients with IXT.

## Introduction

Strabismus is one of the most common diseases in the field of ophthalmology, with a prevalence rate between 0.5 and 5% (1). There are many types of strabismus, which can be roughly divided into exotropia and esotropia. Exotropia is divided for congenital exotropia, concomitant exotropia, non-concomitant exotropia and consecutive exotropia. Among them, concomitant exotropia consists of IXT and constant exotropia (2). Intermittent exotropia (IXT) is the most common subtype of strabismus, with a prevalence ranging from 0.12 to 3.9% worldwide (3–5). IXT is a subtype of comitant exotropia that falls between exophoria and constant exotropia, which manifests as intermittent squinting outward during distance fixation or inattention. The most prominent characteristic of IXT is that the oblique angle of view changes greatly and still having part of the ability to control the exodeviation. It can impair social interactions and lead to psychological problems (6–8).

The etiology and underlying pathological mechanisms of IXT remain unclear making clinics frustrated to choose optimal treatment. Although ophthalmologists chose to use the surgery to adjust the extraocular muscle, the treatment effect was unsatisfactory. Some patients have poor prognosis, such as under correction and over correction. Neurology and pathology studies have proposed that IXT is an abnormality of the central nervous system that is associated with defective binocular fusion (9, 10). Completely developed binocular fusion is crucial for ocular alignment. In humans and other primates, sensory signals from each eye remain separate until they arrive at the primary visual cortex (V1). Dougherty et al. discovered that the signals from both eyes converge and fuse in the input layers of V1 (11). Problems with fusion in binocular vision leads to an inability to form normal stereoscopic vision. Given that V1 cortical neurons encode both detailed stimulus features and eye-of-origin information, the V1 is thought to play a critical role in the detection and resolution of interocular differences (12–14), spatial frequency, and orientation (15–18), which are crucial for normal stereoscopic vision. The disruption of binocular visual signal correspondence in patients with IXT may be due to an alteration of the binocular activation of V1 cortical neurons (19).

Although many researchers have investigated V1 functional changes in strabismus, studies of patients with IXT are rare. In primate experiments, Zhang et al. modeled strabismus in monkeys over 3 days and found a striking increase in the prevalence of V1 neurons that exhibited binocular suppression, where binocular responses were weaker than monocular responses (20). Similarly, other primate experiments using electroencephalogram have also shown that the number of binocular V1 neurons and their bilateral horizontal connections are reduced in strabismus (21–25). However, animal experiments cannot fully represent brain changes that occur in human cases of strabismus and these studies focused on the broad category of strabismus, which cannot represent the brain function changes of specific subtypes of strabismus. Resting-state functional magnetic resonance imaging (fMRI) objectively measures the brain activity and functional connectivity (FC) of specific regions. Zhu et al. observed FC changes between the V1 and left lingual gyrus, cerebellum, right middle occipital gyrus, left precentral gyrus (PreCG), bilateral postcentral gyrus (PosCG), and right inferior parietal lobule (IPL) in patients with concomitant exotropia (7). As a subtype of concomitant exotropia, we speculate that IXT would also show changes in brain function. Furthermore, because patients with IXT are in a transition state toward constant exotropia, still having part of the ability to control the exodeviation with fusion mechanisms, and thus retain partial control of ocular position (26), they may have FC patterns that are distinct from comitant exotropia or other type of strabismus. To date, there have not been any such investigations.

Li et al. found abnormal brain activity in binocular fusion-related cortices in IXT patients using task-based fMRI (2). As for the brain function studies of other types of strabismus, the previous studies mostly focused on patients with concomitant exotropia, and some studies also focused on infantile esotropia (27–29). In patients with concomitant exotropia, the Reho value was increased in the right inferior temporal gyrus, right lingual gyrus and bilateral cingulate gyrus, which Huang et al. believed it indicated that patients with concomitant exotropia had brain function compensation for fusion function (29). In patients with congenital concomitant exotropia, ALFF values decreased in bilateral middle frontal gyrus and increased in bilateral posterior cerebellar lobe and left angular gyrus (28). In infantile esotropia, Yang et al. found increased Bold signals in the left cingulate gyrus, bilateral precuneus, and left angular gyrus (27). However, different subtypes of strabismus have various etiology, pathological mechanism, and clinical characteristics. These studies of strabismus in the general category do not represent changes in brain function in each subtype. As mentioned above, the most prominent characteristic of IXT is that the oblique angle of view changes greatly and still having part of the ability to control the exodeviation. We think IXT must have a unique pathogenesis and brain function changes. Based on previous studies of strabismus, we hypothesized that patients with IXT would exhibit aberrant and characteristic FC patterns between the V1 and other brain areas. The purpose of this study is to find out whether the FC between V1 and the brain regions related to fusion function, stereoscopic vision and eye movement have changed, and to demonstrate the hypothesis of defective fusion mechanism is abnormal by using fMRI. In addition, we tested whether abnormal FC patterns were associated with clinical features of IXT, such as duration and Newcastle Control Test (NCT) score.

## Materials and Methods

### Subjects

Twenty-six patients with IXT (14 men and 12 women, age: 28.23 ± 8.13 years) were enrolled in the study. In addition, we recruited 21 HCs (10 men and 14 women, age: 28.14 ± 5.79 years) from the local community. HCs were matched with IXT patients for sex, age, education, and handedness. All participants underwent detailed ophthalmological examinations that included measurement of best corrected visual acuity, fundus examination, synoptophore, alternate cover test and NCT. The study was approved by the medical research ethics committee and institutional review board of Capital Medical University, Beijing Tongren Hospital, and written informed consent was obtained from all participants.

The inclusion criteria for IXT patients were as follows: (1) diagnosis of IXT based on medical history and clinical examination (loss of stereoscopic vision detected by synoptophore and/or deflection time accounted for more than half of waking time, and exotropia deviation < −15Δ); (2) over 18 years of age; (3) best-corrected VA ≥ 1.0; (4) no medical treatment for any condition received for IXT; (5) ability to understand and cooperate during an examination; and (6) able to voluntarily provide written informed consent. Participants were excluded if they had any of the following: (1) ocular disease (e.g., amblyopia, cataract, glaucoma, optic neuritis, or macular degeneration); (2) other kinds of strabismus, such as constant exotropia, esotropia, or incomitant strabismus; (3) previous eye surgery; (4) vertical strabismus; (5) history of psychiatric, cardiovascular, or neurological condition; (6) drug or alcohol addiction; and (7) contraindication to MRI examination (metal in cardiac pacemaker or prosthesis, or previous head or spinal trauma requiring neurosurgery).

The inclusion criteria for HCs were: (1) best-corrected VA ≥ 1.0 and free of any ocular diseases; (2) no deformities in the brain parenchyma diagnosed by cranial MRI; (3) no current psychiatric condition; and (4) capable of undergoing an MRI examination.

### MRI Data Acquisition

A 3.0-T MRI scanner (Discovery MR750; General Electric, Milwaukee, WI) with an eight-channel phased array coil was used to acquire the MRI data. Earplugs and foam padding were used to reduce scanner noise and head motion. Resting-state fMRI data were obtained using an echo planar imaging pulse sequence with the following parameters: repetition time (TR) = 2,000 ms; echo time (TE) = 30 ms; field of view (FOV) = 240 × 240 mm; flip angle = 90°; slices = 36;thickness = 3 mm; gap = 0 mm; matrix = 64 × 64, voxel size = 3.75 × 3.75 × 4.0 mm, and 180 time points. The total scan duration of the fMRI session was 400 s. A 3-dimensional brain volume sequence was used to acquire high-resolution structural images [TR = 8.16 ms, TE = 3.18 ms, inversion time (TI) = 450 ms, flip angle = 12°, matrix = 256 × 256, thickness = 1.0 mm without gap, 188 slices and voxel size = 1 × 1 mm × 1 mm]. The total scan duration of the 3-dimensional brain volume sequence was 259 s. During scanning, participants were asked to remain still, stay awake, and to not think of anything specific.

### Data Preprocessing

Preprocessing was carried out using Data Processing Assistant for Resting-State fMRI (DPARSF 2.1; State Key Laboratory of Cognitive Neuroscience and Learning, Beijing Normal University, Beijing, China; available in the public domain at http://restfmri.net/forum/DPARSF) and CONN functional connectivity toolbox (30) based on Statistical Parametric Mapping (SPM12) (http://www.fil.ion.ucl.ac.uk/spm/) running under MATLAB R2013b (The MathWorks, Natick, USA). The DICOM files were converted into NIFTI images. The first 10 volumes when participants were adapting to the scanner noise were removed. The images then underwent slice-timing correction, head motion correction, spatial normalization to the Montreal Neurological Institute template (resampling voxel size = 3 × 3 × 3 mm). To reduce the effects of linear tendency due to the long duration of the scan, linear drift was removed from the data. The BOLD signal in resting state is very susceptible to noise, and it is necessary to further remove noise interference by regression Common noises requiring regression include: cerebrospinal fluid signals, white matter signals, head movements, physiological signals, whole brain signals, and so on. Then, the regressed images were smoothed with a Gaussian kernel of 6 mm full-width at half-maximum to remove spatial noise. Finally, a temporal bandpass filter (0.01–0.08 Hz) was used to minimize the effects of low-frequency drift and high-frequency noise. The data were transformed using Fisher r-to-z transformation to improve normality of the correlation coefficients. Data were excluded if there was head motion ≥ 2 mm in any direction or if there was an angular rotation ≥ 2°. None of the data was excluded.

### Definition of the Region of Interest

Both sides of the V1 region (BA17) were chosen as region of interests (ROIs) and created using DPARSF 2.1 (http://restfmri.net/forum/DPARSF). The seed ROIs were defined by the left and right BA17 locations on the Talairach Daemon BA atlas (31). Then the ROIs were normalized spatially to the Montreal Neurological Institute (MNI) space.

### Statistical Analysis

#### Functional Connectivity Analysis

FC analysis during resting state was carried out using the CONN toolbox. FC analyses were performed separately for the left and right V1. A seed reference time series for each hemisphere of the V1 was obtained by averaging the fMRI time series of all voxels within the area. A two-sample, two-tailed *t*-test was performed to compare the V1 FC maps between IXT patients and HCs. The statistical threshold was set at a voxel level of *p* < 0.001 and a cluster level of *p* < 0.05, Gaussian random field (GRF)-corrected.

#### Statistical Analysis of Clinical Data

All non-voxel-wise demographic and clinical data were analyzed with statistical software (SPSS 20 version 20.0.; SPSS, Inc., Chicago, IL, USA). To examine the demographic data, clinical features, and head motion parameters, a two-sample *t*-test was used for normally distributed data, and the Mann–Whitney U test was used for non-normally distributed data. Statistical significance was set at *p* < 0.05.

#### Correlation Analysis Between Functional Connectivity and Clinical Features

We collected data on clinical features, which included duration of IXT, NCT score, and best-corrected VA of each eye. A Pearson correlation was used to examine associations between mean FC values of various brain regions and clinical scores in the IXT patients using statistical software (SPSS 20 version 20.0.; SPSS, Inc., Chicago, IL, USA). Statistical significance was set at *p* < 0.05.

## Results

### Demographics and Clinical Features

Clinical characteristics and head motion parameters of the 26 IXT patients and 21 healthy controls (HCs) are listed in Table 1. Group differences in sex were using the χ^2^ test and the group differences in age, education, the right/left best-corrected VA, and the head motion parameters were using two sample-*t* tests, respectively. There were no significant differences between the groups in age (*p* = 0.967), sex (*x*^2^ = 0.180, *p* = 0.671), education (*p* = 0.726), handedness, or best-corrected VA (right: *p* = 0.792; left: *p* = 0.379). Head motion parameters did not differ significantly between groups (*p* = 0.291).

**Table 1:** Demographics and clinical measurements of IXT patients and HCs.

	**IXT patients (*n* = 26)**	**HCs (*n* = 21)**	***t*-value**	***P*-value**
Sex, male/female	14/12	10/11	N/A	0.671^†^
Handedness	26R	21R	N/A	N/A
Age (years)	28.23 ± 8.135	28.14 ± 5.79	0.042	0.967^‡^
Education (years)	15.38 ± 2.93	16.95 ± 2.72	−1.484	0.726^‡^
Duration (years)	10.33 ± 9.12	N/A	N/A	N/A
Newcastle control test	5.25 ± 1.87	N/A	N/A	N/A
The best-corrected VA(R)	1.02 ± 0.07	1.04 ± 0.13	−0.792	0.379^‡^
The best-corrected VA(L)	0.98 ± 0.11	1.04 ± 0.03	−0.029	0.865^‡^
Head-motion (Mean fd-power)	0.10 ± 0.03	0.11 ± 0.04	−1.070	0.291^‡^

*Data are presented as mean ± SD; HCs, healthy controls; IXT, intermittent exotropia; N/A, not applicable*.

† *χ^2^ test*.

*Two-sample t-test*.

### Altered FC of the V1 Region in Patients With IXT

**Figure 1:**
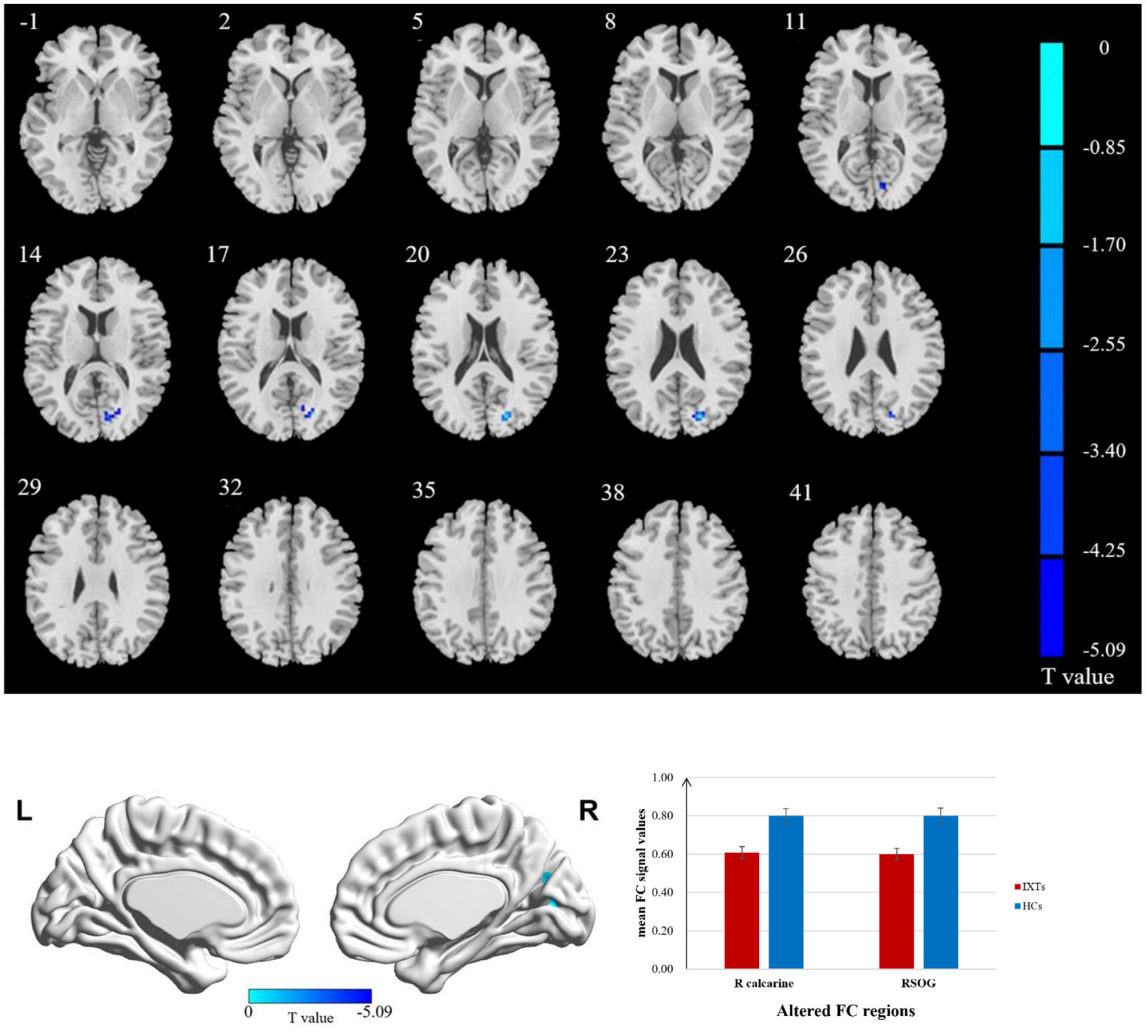
Brain regions demonstrate statistically significant differences between two groups in terms of FC in the right BA17 (GRF corrected, *P* < 0.05). Decreased FC values were observed in the right calcarine and right superior occipital gyrus (SOG). The details of the negative regions can be found in Table 2. IXTs, Intermittent exotropia; HCs, Healthy controls; BA, Brodmann area; FC, Functional connectivity.

**Figure 2:**
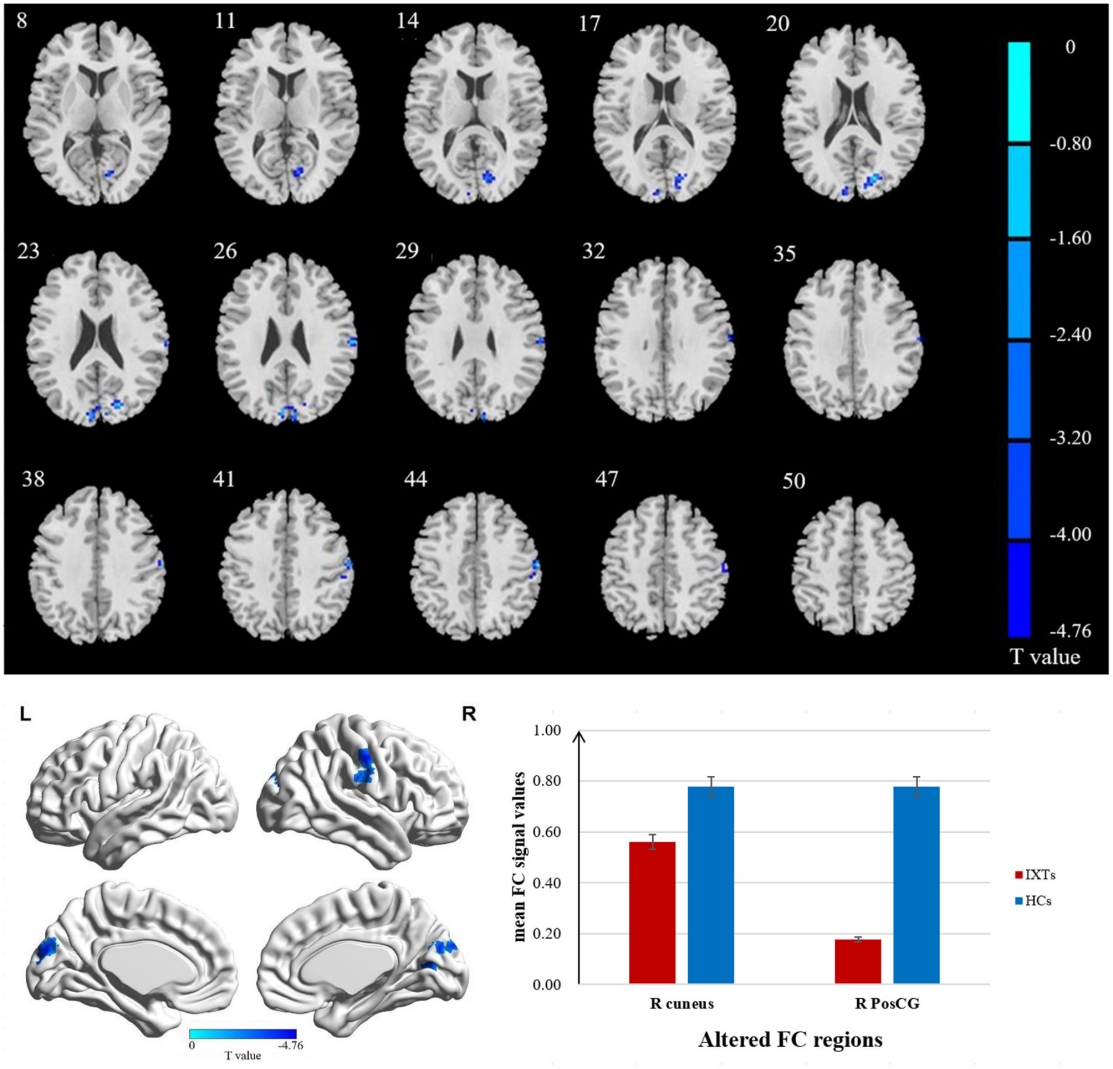
Brain regions demonstrate statistically significant differences between two groups in terms of FC in the left BA17 (GRF corrected, *P* < 0.05). Decreased FC values were observed in the right cuneus and right postcentral gyrus (PosCG). The details of the negative regions can be found in Table 2. IXTs, Intermittent exotropia; HCs, Healthy controls; BA, Brodmann area; FC, Functional connectivity.

The FC values of the right and left V1 [Brodmann Area (BA) 17] to other brain areas that showed significant FC alterations in IXT patients are shown in Table 2. Compared with HCs, IXT patients showed significantly lower right V1 FC with the right calcarine and right superior occipital gyrus (SOG) (Figure 1). There was also significantly lower FC in IXT patients between the left V1 and right cuneus and right PosCG (Figure 2). No increased FC between the bilateral V1 and other brain regions were found.

**Table 2:** Regions revealing significant FC differences between IXT patients and HCs (*P* < 0.05, corrected for GRF).

**Brain region**	**Peak MNI, mm**			**Peak *T* value**	**Cluster size, mm3**
	**x**	**y**	**z**		
**Right BA17**					
R calcarine	21	−78	21	−5.0925	39
R superior occipital gyrus	21	−78	21	−5.0925	39
**Left BA17**					
R cuneus	21	−78	21	−4.7617	89
R postcentral gyrus	60	−15	45	−4.6978	40

*The significance level was set at voxel level P < 0.001 and cluster level P < 0.05, Gaussian random field theory corrected*.

*R, right; L, left; BA, Brodmann area; MNI, Montreal Neurological Institute*.

### Correlation Analysis Between Brain Function and Clinical Features

The NCT score was positively correlated with mean FC values between the right V1 and the left IPL (*r* = 0.473, *p* = 0.035). There was also a positive correlation between the NCT score and mean FC values between the left V1 and the left IPL (*r* = 0.489, *p* = 0.029) and left supramarginal gyrus (*r* = 0.466, *p* = 0.038). The duration of IXT was positively correlated with mean FC values between the right inferior occipital gyrus (IOG) and right V1 (*r* = 0.457, *p* = 0.043). There was no significant correlation between mean FC values and best-corrected VA of either eye in the IXT patients (*p* > 0.05).

## Discussion

In this study, IXT patients had lower FC of the bilateral V1 with fusion function, stereoscopic vision, and oculomotor-related brain regions, including the right calcarine, the right SOG, right cuneus and the right PosCG. The NCT score was positively correlated with strength of FC of the V1 with the left IPL, left supramarginal gyrus. Moreover, duration of IXT was positively correlated with FC between the right IOG and the right V1.

We found that IXT patients showed significantly lower FC between the right V1 and the right calcarine sulcus, which was related to the dysfunction of fusion. The V1 comprises the calcarine sulcus and receives and transmits information directly from the ipsilateral geniculate nucleus along the dorsal and ventral streams, which are essential for the development of normal vision (32, 33). Hubel and Wiesel suggest that sensory fusion occurs in Panum's area of the retina via the action of binocular neurons in the V1 and secondary visual cortex (34). The binocular fusion function is the ability of the brain to translate information about disparities between the images in both two retinas into a vergence command to facilitate stereopsis, including sense fusion and motor fusion, which is a complex cerebral activity. One previous study revealed that binocular inhibition is noted much more frequently in patients with IXT than in the normal population (41.7% compared with 2%) (35, 36). Presence of binocular inhibition in a patient with IXT may indicate diminishing fusional control. Ahn et al. presumed that cortical suppression may be triggered even during fusion in patients with exotropia (26). The lower FC between the V1 and the calcarine sulcus in patients with IXT may indicate an impairment in the interactions within the V1, which may be related to binocular fusion dysfunction. In turn, this may disrupt the coordination and balance of the two visual axes and leads to deviation of the eyes (27), which conformed that the dysfunction of fusion is one of the possible mechanisms of IXT.

Our finding of decreased FC between the left V1 and right cuneus in IXT patients was related to the abnormality of stereoscopic vision, eye movement and emotion. Stereoscopic vision uses binocular differences to extract depth information from two-dimensional retinal images (27). As well all know, the V1 receiving inputs from both eyes is the first step of stereoscopic vision processing (37). The cuneus plays an important role in modifying and transmitting visual information to the extrastriate cortex, which is involved in spatial processing (38). In patients with IXT, stereoscopic vision is damaged to varying degrees. Damage to the cuneus may relate to the deficits of stereoscopic vision observed in strabismus (39–41). One study reported impairments in the structure and function of the cuneus in adults with strabismus, based on reductions in gray matter volume (GMV) of bilateral cuneus (42), which is consistent with our studies. The decreased FC between V1 and cuneus implies that the change of its function may affect the formation of normal stereoscopic vision. The cuneus also contributes to the perception of facial emotion, which is important for social interaction (43) and may explain the social dysfunctions seen in some patients with IXT. Research by Schraa-Tam et al. demonstrated that the cuneus is involved in eye movement reflex that functions to stabilize the image of the retina (44).

In addition to abnormal FC between V1 and cuneus, the FC between the right SOG and right V1, the left V1 and right PosCG were shown to be decreased in our IXT patients, which were also related to eye movement. If the input signals from binocular vision cannot be converted into appropriate eye movement signals, dysfunctional visual perceptual eye movement regulation may result, which may underlie the continuous progression of the disease. The SOG, located in the main visual function area of the parietal eye field, is thought to be associated with saccades (45). Recent behavioral studies in humans and monkeys have reported that strabismus involves disordered patterns of directional and amplitude disconjugacy of saccades (45). Chan et al. found that the GMV of the SOG was smaller in adults with strabismus (42). The PosCG is part of the motor and sensory network and is associated with oculomotor processing. Wang et al. found that the frontal and parietal regions, including the PreCG, PosCG, are associated with spontaneous activity in the V1 (46). Similar results have been reported by Nir et al., who found that fluctuations in resting blood oxygenation level dependent (BOLD) signals in the occipital visual areas were highly correlated with those of the PreCG and PosCG (46, 47). Reduced FC between the SOG, PosCG, cuneus and V1 may interfere with oculomotor disorder in IXTs. In patients with IXT, although, degeneration and fibrosis of the extraocular muscles have been observed with light microscopy and electron microscopy (48), neuronal activity within the oculomotor brain regions plays an important role in eye movement.

The NCT score represents the severity of ocular position control impairment. We found a positive correlation between NCT score and FC of both the left and right V1 with the left IPL and the left V1 with the left supramarginal gyrus, which suggests that the higher the severity of the disease, the stronger the FC between these regions. The IPL and supramarginal gyrus play important roles in the dorsal pathway, which processes spatial position and eye movement. Yan et al. found that the GMV and fractional anisotropy values of white matter fiber tracts were abnormal in the dorsal visual pathway in comitant exotropia patients (49). In IXT, patients retain some ability to control eye position before developing constant exotropia, which may be because of a compensatory process within these brain regions.

The duration of IXT was also found to be positively correlated with FC between the IOG and right V1, which indicated that as the disease progresses, the FC between the V1 and IOG becomes stronger. The IOG participates in the initial stages of visual processing (50). Previous studies have shown that through extensive practice of challenging visual tasks, brain plasticity can be induced in adults with amblyopia (51). We suggest that these FC changes in patients with IXT might reflect cerebral plasticity over time, as they “learn” to use these aberrant regions.

Given that the V1 initiates the processing of binocular fusion, functional abnormalities of the V1 will inevitably affect the formation of normal binocular vision. As mentioned earlier, binocular vision and eye movement are impaired in IXT patients. In our study, we found that FC between the V1 and visual and oculomotor regions were disrupted in IXT patients, which confirmed our hypothesis that these changes are related to the pathogenesis of IXT. Furthermore, we observed plasticity in specific brain regions as the course of the disease progressed, that may allow patients with IXT to develop their ability to control ocular position.

## Limitations

The current study has several limitations. First, the sample size of the study is not very large, studying a larger sample would be useful to allow more detailed neurophysiological and neuroimaging investigations. Second, the present study only explored the FC differences based on the ROIs, in the future, advanced approaches (independent component analysis, network analysis and so forth) are needed to better specify the underlying mechanisms of IXT. Third, we did not collect neuropsychological data from our patients, which may be related to brain regions implicated in IXT.

## Conclusions

IXT patients exhibited decreased FC between the V1 and visual, and oculomotor regions, which are associated with the impaired fusion, stereopsis, and deviation of eye position. These findings extend our current understanding of the neuropathological mechanisms underlying visual and oculomotor impairments in IXT.

## Data Availability Statement

The raw data supporting the conclusions of this article will be made available by the authors, without undue reservation.

## Ethics Statement

The studies involving human participants were reviewed and approved by the medical research ethics committee and institutional review board of Capital Medical University. The patients/participants provided their written informed consent to participate in this study.

## Author Contributions

All authors listed have made a substantial, direct and intellectual contribution to the work, and approved it for publication.

## Conflict of Interest

The authors declare that the research was conducted in the absence of any commercial or financial relationships that could be construed as a potential conflict of interest.
